# New approaches to housing complexity: designing dwellings in the age of cognitive economy

**DOI:** 10.1186/s40410-020-00128-5

**Published:** 2020-11-19

**Authors:** Francesco Spanedda, Matteo Carmine Fusaro

**Affiliations:** Department of Humanities and Social Sciences, University of Sassari, Via Roma 143, Sassari, Italy

**Keywords:** Architectural design, Housing, Dwelling space, Sharing economy, Information capitalism, Cognitive economy, SoHo, ICT, Airbnb, Network society

## Abstract

Space is produced by a society in accordance with its habits, and habits, mostly in the Western society, have been heavily influenced by forms of production. Indeed, it is widely acknowledged that the Industrial Revolution definitely changed the space of modern landscapes, cities and dwellings life at all scales, and the way in which we perceive them. Cognitive capitalism is no exception. Since it fully established itself as one of the prevailing economic forces in the 21st century and in the Western world, it produced deep changes in the way in which people work, connect, and live. Starting from the assumption that changes in means of production generate new social relationships, this paper investigates how these changes might result in new ways of building architectural space. Without indulging in a deterministic attitude, it focuses on housing as one of the fundamental artefacts where a society expresses its approach to space. The house is a basic element of complex urban systems and is, therefore, the one calling for a more radical conceptual rethinking, marking an effective distance with the forms inherited from the previous centuries. Finally, the paper aims at understanding the repercussions of the digital paradigm on the space of dwelling, reasoning on some crucial questions to understand how housing might evolve, unfolding through its spatial configuration the new ways of life of the digital society.

## Introduction: dwelling in a time of change

Houses are among the most important constitutive elements of the cities. They could be viewed as physical structures that at the same time define a subsystem of the environment and a multi-faceted set of smaller spaces which support the life of a single family or a group (Coolen and Meesters 2012). As a matter of fact, the spatial organisation of the house reflects and influences in more or less direct ways how the inhabitants interact among them and with the external world.

In spite of the deep societal changes in the last decades, their spatial organisation still relies on the typologies established during the late 19th century and the beginning of the 20th century (Kahler 2002), when the Second Industrial Revolution introduced radical changes in several aspects of everyday life, such as labour organisation, population distribution, wealth distribution, hygienic standards, building technology, and eventually in individual habits.

Instead of offering new typologies, the housing market currently fulfills the requests of contemporary society mostly by technology upgrades. As an example, environmental concerns result in a wider production of energy-efficient housing by largely improving the performance of building envelopes and services, while financial limitations are met by low-cost building systems.

However, just as in the past the shift to industrialism led to new ways to conceive modern housing, one can wonder if contemporary needs are calling for—if not introducing already—deeper spatial changes than just technological ones.

Therefore, this paper draws from Castell’s (1996) statement that all major social changes are ultimately characterized by a transformation of space and time in the human experience, and investigates actual and potential changes in the dwelling structure sparked off by the transition from industrial production to the production of information.

A further point is Wark’s (2004) assertion that every change in the means of production brings to the emergence of new classes. One could therefore expect that the transition between the *fossil paradigm* at the core of the Industrial revolution and the current *digital paradigm* might deeply influence today’s society and most probably its housing since the history of dwelling shows how different forms of social stratification co-evolved with different housing types, producing several ways of living in cities and homes.

Just as modern housing was primarily an answer to the needs of the industrial working class—a circumstance that much irritated Tom Wolfe (1981), outraged by socialist aesthetics spreading among the north American elite—might contemporary housing evolve together with the diffusion of what Wark calls the hacker class, and of the other kinds of workers in the digital economy?

Purposely leaving in the background environmental and financial issues, which also question the traditional spatial organisation of houses, this paper tries to detect the traces and evidence of change in housing, induced by the new ways in which production influences everyday life.

## Reducing complexity: housing in the Second Industrial Revolution

At the beginning of the Second Industrial Revolution, architecture started to cope with the housing crisis throughout Europe.

The change in production methods established a discontinuity with the past, both in the localisation of production sites, since fossil fuels allowed to locate factories far from watercourses, windy hills or other energy sources, and in the organisation of time, because working hours became independent from seasons and the availability of natural resources. Peasants became workforce, and had to leave the countryside to move closer to the factories. Thus the new means of production, by changing the working conditions, structurally altered the relationship between people and places, clearing the way for deep changes in culture and habits.

These changes directly concerned urban planning and architecture. The most important European cities dealt with these emerging issues by shifting from the 19th-century city to the 20th-century metropolis, in a process that developed both at the scale of the city and at the scale of the dwelling.

The first radical change was the definitive separation of *work* and *life*, in contrast to the pre-modern household, a spatial structure that organises the greater part of the activities needed to survive by integrating work and family life. In farms or workshops, the whole family and its generations, when not different families, participate in the daily work without any actual separation between family care and labour, neither in space, nor in time (Junestrand and Tollmar 1998).

The establishment of factories disrupted this substantial continuity. Work and labour were no longer a part of the house, but they found appropriate and well-organised spaces inside factories and offices. Time split along with space, as coordinating the effort of workers living in different places required the scheduling of activities in a part of the day exclusively devoted to work.

Houses thus became a place dedicated to the private sphere: personal needs, spare time activities, and child care.

Under the pervasive influence of Taylorism, hygienic issues and equalitarian aspirations were mainly solved through “the gradual transformation of life into economy and production” (Aureli 2014). Domestic space was “no longer conceived as just the symbol of kinship and family bonds, but as an infrastructure devoted to the reproduction of life”, which eventually resulted in houses becoming housing, that is the “functioning of the house” (ibid. p. 30).

Thus, housing becomes just a piece of the urban infrastructure, losing its specificity and achieving a status similar to that of a machine. The need arises to define the qualities of this new product, that for the first time comes to be an object of mass production.

Housing becomes at once a physical infrastructure and a political construction when its spatial arrangement frames the role of a group within the new society. Social housing especially becomes the representation of a paternalistic relationship between employees and employers, or a tool of emancipation as the basis of welfare state.

To define the character of the new living spaces, Modern architects deployed two apparently opposite spatial strategies.

The first strategy revolved around Taylorism, and its implementation into the design process. Notable examples are Le Corbusier’s *Ville Radieuse*^1^ (1935), Alexander Klein’s studies on the dwelling layout (1934), and Margarete Schütte-Lihotzky’s *Frankfurt Kitchen*^2^ (1926), respectively at the scale of the city, of the building, and of the room and furniture. All of them translate the Tayloristic sequence of analysis, functional separation, and optimisation into the design of cities and dwellings. The whole city, the house, the rooms themselves, take the organisation of a factory. The demarcation between each function is clear without any overlap, the paths do not cross, aisles and corridors are reduced to the minimum.

These proposals provide sharply defined, meticulous, and beautiful line drawings that are at once analytical tools and ways to communicate their findings. This graphical apparatus shows how the reductionist approach of Taylorism leaked out of the design process of housing to become an organisational model for the life of the family occupying it.

Dwellings became thus one of the ways in which Taylorism spread out of factories to become a pervasive organisational model for every part of the society, not limited to factories for workers and office for employees.

The second strategy promoted flexibility, a concept which actually underpins two different design strands.

The first strand ideologically exploits the technological innovation of structural concrete, conceiving dwellings as the stacking of free floors, in a spatial arrangement similar to that of factory buildings and turning housing, and therefore the city, in an extended factory. A clear example is the 1914 Le Corbusier’s *Maison Dom*-*ino*^3^ (Aureli 2014, p. 30), where the radical image suggested by the standardised grid of floors and pillars is balanced by allowing the owners to freely configure its facades.

The other strand invokes flexibility as an antidote to standardisation and its spreading outside the factory. In his text about the *Woman as creator*, Bruno Taut firmly expresses his contrariety in drawing floor plans so that “a living room is meant for living, and sleeping cabins for sleeping—and for nothing else… Rather in the manner of an engineer who regards the standard family with three children as an undertaking for which he is constructing the machines and the factory” (Taut 1924). In opposition to this, Taut states that families do not follow any organisational model, but are the creative outcome of the work of housewives, whose constant effort deals with very specific conditions. Therefore, to allow a family to freely organise its life, a dwelling needs just rooms of different size, whose function and relationship will be decided by the occupants. Taut’s work explicitly criticises the determinism of the Tayloristic approach, even in its attempts to improve the housewife’s life by imposing an organisation model, like in the case of Lihotzky’s *Frankfurt Kitchen*.

With its emphasis on hygiene, comfort, privacy, authonomy, and eventually consumption, the modernist idea of dwelling became so pervasive that currently most literature, especially in the West, overlook the concoction of work and family life in the domestic space for a long time before Industrialism, and that this commixture has been kept in rural areas until recent times. Also, it is worth to remember that 90% of the dwellings on the world are not of the single-family type (Oliver 2003). Thus, the separation of functions, spaces, and even between the family members is not something that has to be taken for granted. According to Meloni (2014), home boundaries are porous by nature: they establish connections with the external world and with different times, by opening views onto the landscape, collecting the memories of their inhabitants, and accommodating friends and sometimes strangers. This potential comes again to the fore in the digital age, when houses become the hubs of a networked life (Miller 2008).

## Back to home: the age of cognitive economy

In the 1990s information technology began to heavily contribute to industry’s productivity increases. From that time on, the rise of the *knowledge economy* and then of *cognitive capitalism* in its different forms established a break with the industrial tradition. The production of value moved from actual products to immaterial, abstract activities of data gathering, of organisation of information, of decision-making, and finally of control.

Although the elaboration of knowledge and the collection and control of data accompanied industrial capitalism from its beginnings, two main factors boosted them significantly. On one hand, the spreading of IC technologies and computers, which are universal machines that rapidly became constitutive parts of the production process, and their ubiquitous networks that can operate globally and in real time (Schmiede 2006; Rasmussen and Corbett 2008). On the other end, the diffuse intellectually, the mass education, and the high level of training produced by the welfare state in late post-war time (Vercellone and Lucarelli 2013).

Hence the various definitions as *informational capitalism* (Castells 1996) or *vectorialism* (Wark 2004).

At the same time, factories shut down all over Europe and the US since the late 1960s and 1970s, (Florida 2005) showing how industrial capitalism was losing economic power, while a new form of production, strictly connected with technological progress, was gaining traction: the *Innovation Industry* (Moretti 2014).

Among the main consequences of the dematerialisation of production, is the loss of importance of material capital in form of factories, buildings, large equipment, and the acknowledgment of the increasing value of the intellectual capital of a company, embodied in human beings and in their relational, emotional and cognitive faculties.

Along with these distinctive features, IC technologies amplify what Harvey (1989) calls *time*–*space compression*: a condition of postmodernity revolutionising the objective qualities of time and space resulting from the condensation or elimination of spatial and temporal distances—similar to Wark’s (2004 p. 154) *thelestesia*.

*Time*–*space compression* thus annihilates also the boundaries between work, life, and leisure that permeate the modern cities and houses. Wark (2015), therefore, argues that this radical alteration of the parameters of space and time does not just affect production, but also the workers’ way of life.

While *industrial capitalism* considered the house as a safe and clean shelter with clear functions distributed in space and time in accordance with the new organisational doctrines, *cognitive capitalism*, thanks to its higher level of abstraction and its inherent fluidity, offers multiple interpretations of the interactions between working and dwelling.

Instead of producing new typologies, it builds on the housing types inherited from the previous times, adapting them to the pervasive digitalisation and the new types of labour and ways of life.

Although the consequences of this change are widespread and evident, there is still very little literature about them, and even less architectural concepts.

In spite of this, there are some clues to start investigating the architectural consequences of this cultural change, as new forms of organisation necessarily influence the city and its spaces (Cacciari 2004). According to Bernardo Secchi (2013), at every occurrence of a shift in the relationship between capital and labor the city suffered an urban crisis, changing on its own, showing resilience and adaptation.

Since the new form of production depends on the intellectual qualities of the workers, the quality of the firm’s material capital, like buildings and hardware becomes less important, and therefore from their localisation in space. Thus, the relationship between workers, their workplaces and their homes changes again. Moreover, the progressive digitalisation offers new opportunities of commodification. The rental market, traditionally limited to local customers, becomes accessible to customers all around the world, accessible from almost everywhere at a reasonable price thanks to low-cost flights.

The change unfolds along several major trends: the return to of integration between house and workplace, similarly to the pre-modern era, now called SoHo (Small office/Home office); the use of housing as benefits to attract new employees and dampen the intrinsic precarity of the new low-term jobs, feebly echoing the paternalism of industrial capitalism; the transformation of housing in a palatable commodity that, in spite of its localisation, finds a worldwide market thanks to the digital economy; the swapping of the public and the private sphere between the interior and the exterior of the house.

### Living rooms/working rooms

Information and communication technologies played a key role in the transformation of production and ultimately of labour.

The abstract, virtualised new production does not require proximity in space and time anymore. Instead, employees are gradually turning into *digital nomads*, able to work “anytime anywhere” (Roberlski, Keller, Harth, Mache 2019). This opportunity is not offered just to creative freelancers traveling all across the world, but to a greater crowd of white collars, which can work from home *telecommuting* thanks to the massive presence of *ICT* in the domestic environment. The so-called *telework*, as defined in 2002 by European Social Partners, can be defined as a transposition of the traditional way of working into the virtual sphere, especially practiced by those categories of workers operating in a different place than their company. *Smart work*, an alternative working method to traditional office work also based on teleworking, allows people to decide how and when they should carry out their duties, beginning with their choice of space, time, programs and tools.

This *modus operandi* also fits perfectly with the needs of the new generations of workers who are entering the labor market, the so-called *digital natives*. Although *digital natives* are able to work wherever there is a Wi-Fi connection available, especially in those places where they can *network*, like co-working spaces, the house is rapidly becoming, at least for some social groups, a place fully equipped with ITC.

In spite of this, a difference remains between *working from home* and *working at home*. Regus, a leading company in flexible workspaces all over the world, conducted a study on the emergence of *smart working*. The sample of respondents highlighted as problems the requests for attention from their family members, a slow or unreliable Internet connection, the difficulties in accessing the main office equipment, the presence of background noise represented by the family and animals, and the presence of domestic noises.

On the other hand, although part of the work can be done in a non-obtrusive way by exchanging emails and files, video calls, remote conferences, and receiving clients conflicts with the intimacy of the domestic space, which is still mainly built in accordance with the doctrine of functional separation advocated by the design culture of industrial capitalism.

Potentially, the return of work inside the space of the house is a jump back towards pre-modern schemes. However, the different nature of the dematerialised work, and the affirmation of new typologies during the industrial era, both suggest different forms of juxtaposition of family life and work.

Two different trends emerge from the scarce examples of buildings purposely designed around this theme.

At one end of the spectrum is the Jian Wai Soho quarter^4^ designed by Riken Yamamoto & Field Shop in Beijing (2004). The spatial concept envisions a cluster of towers, whose apartments feature a *SoHo* area between the most private part of the house and the hallways, freely accessible and configured as a pedestrian public space. The resulting space is a combination of a mall, given the public character of the passages and the shop windows of the SoHo rooms, and an apartment tower made of rather usual dwellings, whose rooms are dedicated to the traditional functions. Work and family life here do not overlap, but simply juxtapose in order to coexist and interfere as less as possible, formulating a pragmatic, but not groundbreaking, proposal of mixed typology.

At the opposite end of the spectrum is the Pitt Studio^5^ by Graft Architects (1998), a space intended to accommodate a “hybrid lifestyle” where “living and working take place in the same space”, according to the designers. The design concept features a wide surface without any functional subdivision, defined just by its enclosure. Activities inside this apparently blank space are enabled by a central core, a *scripted wall* (with an obvious reference to the profession of the owner, a renowned actor) whose cladding unfolds, rotates and slides sideways to morph into table, seats, lights and niches.

Although the meticulous design of every part and its relationship with the other could resemble the analytical attitude of Klein’s drawing, there is no expectation to build spaces defined by functions, even if temporarily. Each of the movable elements, or each possible combination of them, can activate separate, different activities, like a table can be used for eating or working. Instead of working by juxtaposition, Graft’s spatial experiment interprets the inherent fluidity and openness of the new way of production, offering a stage to a multiplicity of situations.

These two examples show two possible ways to bring together life and work under the same roof. However, they mostly address the needs of freelancers and creatives, which are just a part of the knowledge workers.

At the time of writing, tele-working is receiving a massive boost due to the mobility limitations imposed by the sanitary policies against the Coronavirus pandemic throughout the world. The advantages and shortcomings of white-collar work at home have been amplified, since homes became at once offices, schools, and malls. Whole families are busy with,—or queuing for—remote activities. Undoubtedly based on the tools deployed by cognitive capitalism, this particular condition is more depending on the Coronavirus outbreak than in a change of means of production. There is, however, an increasing trend in allowing the employees to keep working from home (Dwoskin 2020), adducing reasons that oscillate between care of workers’ satisfaction and savings on relocations. However, pandemic is a peculiar case that will be the subject of a different paper within this research.

### Home as a benefit

The delocalisation of production in different places has many other consequences than just being able to work at home. As stated by Moretti (2014), in industrial capitalism, the location of the factories determined the geographical distribution of workforce. Similarly today, the location of innovative companies attracts workforce in the advanced countries (Morrill and Sommers 2005), where the human capital populates some key areas with a high content of creativity. Moreover, not just the material production, but also the immaterial one can be relocated either in places where there is a major concentration of innovative and skilled workers, or where the workforce is cheaper and the overall conditions are economically more rewarding for the employer.

Housing, therefore, gains a new role as one of the benefits that make a position attractive: in addition to its material value, a house also attains a potential value.

This happens at all levels of recruitment: a house and a car are often fringe benefits supplementing a top position, but lodging could also be an added extra for workers with lower wages.

As an example, customer service companies relocate their offices in specific areas of the world, and consequently also look for workers capable of complying with their qualitative standards. As a matter of fact, many call centres do not operate from the nation where the user calls, but in other parts of the world where such kinds of services are mostly concentrated. Nonetheless, the operators answering by telephone, chat or email, should be able to interact perfectly in the customers’ language. As a consequence, companies often recruit workers from the country where the service is being delivered, providing an employment contract that includes some benefits and perks including also a house shared with other colleagues. Although these houses do not seem to be subject to design research, until now, they have a great potential for architectural investigation, bordering the risk of becoming gated communities on one side, and 21st century editions of the factory houses for workers on the other side. Without falling in the reductionist trap of the perfect bubble for knowledge workers, their design can investigate whether a community could be really built around a working environment artificially implanted in a foreign context, which activities can be put in common, if there is a viable opportunity to combine life and work together, and, above all, how such a community can spatially relate with the surroundings.

### Hosts or guests

The American company Airbnb started in 2008 as an online platform to rent empty rooms inside households. Like many other success stories of the *sharing economy*, Airbnb established another way of working. Being an Airbnb host quickly became a full-time occupation for many homeowners in touristic locations, often as an alternative to unemployment or declining liberal professions.

After a slashing success with its digital product, the company raised its ambitions: in 2016, in fact, Airbnb announced the birth of *Samara*, a division tasked with designing and building architecture.

*Backyard* (Samara 2018) is the first initiative of Samara. It aims at investigating how a home designed and built for sharing should look and feel. According to the company, the prototypes should investigate the potential of the space in all its facets. *Backyard* put several timely questions. The key points of the project concern new construction techniques, the use of ecological materials with reduced environmental impact, the reduction of construction waste, the use of *smart home* technologies, self-sufficiency, and the need for cohabitation. *Samara*’s first work in this direction is a prototype house presented at the *House Vision 2* exhibition held in Tokyo and directed by the designer Kenya Hara; through the collaboration between *Samara* and the japanese architect Go Hasegawa, Airbnb has proposed *Yoshino Cedar House,*^6^ (Gebbia and Hasegawa 2016) a housing model that involves the use of local materials, a communal space in the ground floor, and a cooperative hosting by a group of local residents.

Looking at the first built Samara’s house and at the pictures of architectural models on *Backyard*’s site, the whole exploration seems oriented towards flexible and dynamic home models trying to conjugate global hospitality and *genius loci*.

More than in its promising, but still limited achievements, the interest in Samara’s work lies in its reasons. In fact, according to Airbnb, the idea of establishing a design company surfaced when they realised that many of the company’s hosts felt the need of modifying their homes to meet the guests’ expectations. This means that the economic pressure exerted by short-term rentals are much more far reaching than expected. Firstly, they produce a sudden change in roles: by sharing their private space, house owners lose their status and acquire the same rights of their guests, turning themselves into roommates. Secondly, the short-term rentals modify urban occupation patterns, land value, availability, price of long-term rentals, and neighbouring economic activities (Gurran and Phibbs 2017; Picascia et al. 2017).

Finally, the motivation behind Samara reveals that change is propagating at an architectural level, with a sneaky process of adaptation of spaces to new activities.

Whether hosting through Airbnb is just another way of earning from owning an apartment, or a way to enrich own lives and the city by overcoming the equation *stranger *=* danger* (Gebbia 2016) and offer hospitality in the next room, the entity of the transformation taking place in the body of cities and inside the buildings is becoming quite relevant. Therefore, a critical revision of the changes induced by the *sharing economy* in the spatial structure of the house and of the city deserves a collective discussion that cannot be just led by the main economic player involved in it.

### Private goes public

Between the 1960s and 1970s, sociologists highlighted the loss of meaning and importance of public space induced by socio-cultural transformations and ways of life changes. According to Habermas (1962), the public sphere embraces three dimensions: an ephemeral sphere, a physically staged dimension, and an abstract sphere, built by the combination of mass-media and their delocalised audience. Sennett (2017) attributes responsibilities for transforming the concept of public space to economic policies. The transformation of public space results in a gradual loss of traction, while people increasingly retreat within the private domain, which in the meantime is experiencing an unprecedented evolution. In fact, this trend gains momentum due to the increasing role of ICT technologies in expanding the availability of media and of their content, thus powering up the abstract sphere and affecting different areas of everyday life.

Furthermore, the expansion of digital services relocate within living rooms and bedrooms a great amount of the fundamental services for the person, and other activities that usually take part in public spaces: banking, food, shopping. This further shift makes public space *potentially* irrelevant (Harvey 2006).

Advancements in technology transformed living rooms in place of exchange, encounter and confrontation, absorbing the character of the town square in a dematerialised way.

An emblematic case is the sociological phenomenon of the *Second Screen*: sitting in their domestic privacy, people are at the same time spectators of what happens outside their house, but capable of interaction with the events through *social media*.

The home is not just a private space anymore, but an *integrated space* aiming at reconciling every component of contemporary life, the old ones and new ones arising from the *ICT* paradigm.

## Conclusions

The aim of this paper is to provide an overview about the implications on housing design due to the shift from industrial to cognitive capitalism.

The second industrial revolution sparked a great change in housing design. The Modernist legacy is made of a great amount of typological studies of great interest. Most of them show a reductionist approach to architectural design, deeply influenced by the managerial culture of industrial production and based on optimisation, on the definition of functions and their separations. In spite of the strong connection with their time, and of the swift changes that later occurred in economy and society, modern housing typologies are still widespread today.

However, the change to cognitive economy and its related forms of capitalism swept away the linear thinking of industrialism. Networking, real time calculations, telecommunications, allowed complexity to re-emerge in everyday life, disrupting not only the trust in spatial distribution and time schedules of industrial capitalism, but also the possibility of reorganising them again in deterministic terms.

Completely new ways of working and living, like home working, guest hosting, and media production are now laid on spaces that were designed with separation, privacy, and different functions in mind. On the other hand, the use of housing as a benefit in working contracts is reminiscent of the old paternalistic attitude of industrialism, but without the grand aspiration to produce new housing models. Moreover, just like urban space lost part of its public character, housing lost its connotation of privacy and intimacy. Houses are now places open to the countless digital encounters and confrontations, expanding themselves into the urban world into an urban dimension.

The question arises, then, if the notion of typology is still useful in spite of these changes, or if the burgeoning digital environment is getting rid of any classification based on spatial patterns, if not of the whole concept of spatial organisation.

The examples mentioned in this paper, though, show that spatial organisation is still an issue, that space is yet a field of discussion. While the idea of typology as a formally based spatial taxonomy is probably showing its limits, there are other ways to ground a typological discourse that could prove highly useful at the present time.

Drawing on Lévi Strauss (1962), Martí Arís (1993) radically disconnects typology from history, as two complementing parts of a discourse on architecture. While history focuses on transformations, typology investigates permanence. They are thus inextricable, because historical change brings the essence of an object to the fore, and essence becomes a cluster of potentialities available for further development. Arís’ typology is then twofold, as it concerns itself with the existing architectural space but also unveils its future potential.

From a phenomenological point of view, Gregotti (1972) formulates a definition of typology based on spatial relationships more than on form. Such classification locates every architectural organism within a *field of possibilities* depending, on one side, from the tightness of the relationships between its internal components (its *autonomy*), and on the other side, by the quality of its connections with the context (its ability at *establishing relationships* at different levels).

Arís’ epistemological investigations and Gregotti’s operative framework are two different examples of how typology can still overcome a reductionist approach to maintain its role as a designer’s tool.

There is still a great field to explore then, for a critical approach to housing design.

The juxtaposition of typologies and functions, the possibility of thinking dwellings not as a sum of functions but as spaces activated by users—like some Modernist experiences already suggested, the inner flexibility requested by the sharing economy, the design strategies to implant corporate housing inside urban areas, and finally the concept of housing as one of the multiple intersections between the physical and the digital space, are design challenges to bring complexity back as a working material.

Architecture is not probably able to answer all the deep social questions raised by cognitive capitalism, just like Engels did not believe that urban science could contribute to solve the social problems related to the *Great Industry*.

It could however try to advance proposals about organisation, connections, juxtapositions and modification through the space and over time, as a hinge between physical and digital, and at all scales, starting from the indispensable relationship between the city and the house, where changes propagate from one term to the other.

Dealing with the challenges thrown by cognitive economy, contemporary architecture should deal with the task of providing new physical systems of relationship between the spheres of dwelling and those of work, leisure and entertainment.

## Data Availability

All data sources are cited within the text.

## Footnotes

1 Image: http://www.fondationlecorbusier.fr/corbucache/900x720_2049_1975.jpg.

2 Image: https://commons.wikimedia.org/wiki/File:Frankfurterkueche.jpg.

3 Image: http://www.fondationlecorbusier.fr/corbucache/900x720_2049_1513.jpg.

4 Image: http://www.riken-yamamoto.co.jp/cnc_manager/file/project/soho02.jpg.

5 Image: https://graftlab.com/portfolio_page/pitt-studio/.

6 Images: https://www.yoshinocedarhouse.com/the-design.
